# Differentiation between small hepatocellular carcinoma (<3 cm) and benign hepatocellular lesions in patients with Budd-Chiari syndrome: the role of multiparametric MR imaging

**DOI:** 10.3389/fonc.2023.1282181

**Published:** 2023-11-21

**Authors:** Ghazal Zandieh, Haneyeh Shahbazian, Hao Tang, Qingxia Wu, Mohammadreza Shaghaghi, Bita Hazhirkarzar, Azarakhsh Baghdadi, Shadi Afyouni, Franco Verde, Timothy Pawlik, Ihab Kamel

**Affiliations:** ^1^ Johns Hopkins Medicine, Johns Hopkins University, Baltimore, MD, United States; ^2^ The Johns Hopkins Hospital, Johns Hopkins Medicine, Baltimore, MD, United States; ^3^ Department of Surgery, Division of Surgical Oncology, The Ohio State University Wexner Medical Center and James Comprehensive Cancer Center, Columbus, OH, United States

**Keywords:** Budd-Chiari syndrome, small hepatocellular carcinoma, benign hepatocellular lesions, MRI, dysplastic nodule

## Abstract

**Objective:**

To investigate the value of multiparametric MR imaging to differentiate between small hepatocellular carcinoma (s-HCC) versus benign liver lesions in patients with Budd-Chiari syndrome.

**Methods:**

12 patients with benign hepatocellular lesions and 32 patients with small (<3 cm) HCCs were assessed. MRI images were reviewed by two radiologists blinded to the patient background information; lesion T1 and T2 signal intensities and ADC values were compared with the background liver. Enhancement of lesion relative to hepatic parenchyma [(T1_Enh_-T1_liver_)/T1_liver_] in the arterial, venous, and delayed phases was also compared between the two groups. A multivariable logistic model was developed using these categorical measures; the predictive value of the model was tested using the Area Under the Receiver operating characteristic (AU-ROC) curve for logistic models. *P-values <*0.05 were considered statistically significant.

**Results:**

There were consistent differences in T1_lesion_/T1_liver_, and T2l_esion_/T2_liver_, and ADC_lesion/_ADC_liver_ between benign hepatocellular lesions versus the sHCC group (p<0.001, p<0.001, p = 0.045, respectively). Lesion-to-background liver enhancement in the portal venous and delayed phases was different between the benign lesions versus sHCC (p=0.001). ROC analysis for the logistic model that included the T1 ratio, T2 ratio, and portal venous enhancement ratio demonstrated excellent discriminatory power with the area under the curve of 0.94.

**Conclusion:**

Multiparametric MR imaging is a useful method to help differentiate benign liver lesions from sHCC in patients with Budd-Chiari syndrome.

## Introduction

Budd-Chiari Syndrome (BCS) is a hepatic outflow obstruction condition affecting the hepatic veins to the right atrium-inferior vena cava (IVC) junction, divided into primary (P-BCS) and secondary (S-BCS) types, with the latter often due to external factors like malignancy (1, 2). P-BCS typically results from venous thrombosis or phlebitis and is frequently associated with a hypercoagulable state, which has diagnostic and therapeutic significance (3, 4). P-BCS can be further classified by the site of occlusion, either in the hepatic vein or IVC, with hepatic vein occlusion more common in Western countries and linked to hypercoagulable disorders like factor II mutations, factor V Leiden mutations, myeloproliferative neoplasms, and paroxysmal nocturnal hemoglobinuria (5, 6).

The problem is chronic hepatic congestion from BCS may lead to histological changes such as nodular regenerative hyperplasia (NRH), cirrhosis, and large regenerative lesions resembling focal nodular hyperplasia, which can sometimes be mistaken for small hepatocellular carcinoma (s-HCC) (7).

Long-term follow-up studies have indicated that a considerable percentage of BCS patients (up to 17.6% pooled prevalence) develop hepatocellular carcinoma (HCC) worldwide, while the prevalence is reported to be 6.9% in the United States. Factors such as hepatic venous pressure gradient, male sex, factor V Leiden mutation, and IVC obstruction have been proposed as potential risk factors for the emergence of HCC in BCS patients (6, 8, 9). Distinguishing benign versus malignant liver lesions is critical to inform the appropriate treatment of HCC. Differentiating s-HCC from benign hepatocellular lesions in Budd-Chiari syndrome remains a diagnostic challenge, particularly among patients with cirrhosis. However, there is a scarcity of data on multiparametric MR quantitative radiologic imaging specifically aimed at distinguishing benign liver lesions from s-HCC in patients with Budd-Chiari syndrome (6). Consequently, the goal of the current study was to characterize MR imaging characteristics that are distinguishable between benign hepatic lesions and s-HCC in patients with Budd-Chiari syndrome.

## Materials and methods

This was a retrospective study conducted in accordance with the Health Insurance Portability and Accountability Act (HIPAA). The institutional review board approved the study with a waiver of informed consent.

### Study population

Patients with BCS were identified and divided in two groups who had benign hepatocellular lesions (benign hepatocellular lesions group) or s-HCCs (small hepatocellular carcinoma group) and individuals without sHCC. In particular, a systematic database search was performed to identify patients diagnosed with Budd-Chiari syndrome at our institution from February 2005 to June 2018. BCS was defined as any level of hepatic venous outflow impairment between the small hepatic veins and the right atrium (2). The date of the diagnosis of BCS was defined as the initial imaging test that demonstrated obstructed venous outflow. Inclusion criteria were as follows: a) BCS evident on MRI; b) one or more liver lesions seen on contrast-enhanced MRI (CE-MRI); and c) histologic confirmation by percutaneous biopsy or surgery. In the absence of histologic confirmation, stability of size, and imaging features for at least one year, alpha-fetoprotein (AFP) values less than 15 ng/ml were considered evidence of benign hepatocellular lesions (3). Patients were excluded if no hepatocellular lesions were present or MRI was not available. Patients with hepatocellular carcinomas smaller than 3 cm were identified from December 2008 to July 2017. A small (<3 cm) tumor size was chosen to match the size of benign hepatocellular lesions in BCS (10). Malignancy was confirmed by histologic evaluation, iodized oil accumulation after transcatheter arterial embolization therapy, increased serum AFP level that decreased immediately after transcatheter arterial embolization; criteria for HCC diagnosis on MRI included: TlWI hypo-, iso-, or hyperintensity to the liver, T2WI hyperintensity to the liver, and contrast-enhanced Tl arterial enhancement with washout on portal venous and delayed phase (11).

### MR imaging protocol

Magnetic resonance imaging (MRI) was conducted using 1.5 T and 3 T scanners (Magnetom Avanto, Siemens Healthcare, Magnetom Verio, Siemens Healthcare, Signa HDx, GE Healthcare) with a phased array torso coil. The imaging protocol included the acquisition of axial T2-weighted fast spin echo images with and without fat suppression (repetition time ms/echo time ms, 2000–4500/90–100; section thickness, 4–6 mm), axial breath-hold echo-planar diffusion-weighted images (1100–3500/45–75; section thickness, 8 mm; b values 0, 50, 500, and 750 s/mm^2^), and axial breath-hold T1-weighted three-dimensional fat-suppressed spoiled gradient-echo imaging (3.95–5.77/1.35–2.77; field of view, 320–400 mm; section thickness, 2.5 mm) before and after intravenous administration of gadopentetate dimeglumine (Magnevist, Bayer Health Care) at a dosage of 0.1 mmol/kg body weight. The contrast-enhanced scans were performed in the arterial, portal venous, and delayed phases at 20, 70, and 180 seconds after the injection, respectively.

### Image analyses

a radiologists with10 years of abdominal MRI clinical expertise, who was blind to the clinical diagnosis and patient demographics, carried out the image analysis. The reader recorded the numbers and the location of liver lesions. The largest diameter of the lesion (in mm), the signal intensity of the liver lesions, as well as liver parenchyma, and the ADC value of the lesions and background liver were assessed. Signal intensity and ADC value were measured on operator-defined regions of interest (ROIs) that were manually selected using Vue PACS (Carestream Health). ROI of the background liver chosen at least 1 cm in diameter and at the same level as the ROI of the lesion, avoiding vessels and the edge of the liver. Lesion T1 signal intensity (T1SI) and T2 signal intensity (T2SI) to background liver ratios (T1_lesion_/T1_liver_; T2_lesion_/T2_liver_) and lesion ADC value to liver ADC ratio (ADC_lesion_/ADC_liver_) were calculated. Enhancement of lesions relative to hepatic parenchyma [(T1_lesion_-T1_liver_)/T1_liver_] was calculated in arterial, portal venous, and delayed phases.

Statistical analysis was performed using the STATA software (StataCorp, Stata Statistical Software: Release 17, College Station, TX: StataCorp LLC).

Measurements of T1SI, T2SI, ADC, and triphasic relative enhancement maps were compared between the benign hepatocellular lesions and s-HCC groups using univariate and multivariable analyses. Frequencies and percentages were used to convey categorical data, and means, standard deviations, or medians and interquartile range (IQR) were used to represent continuous variables. A Fisher exact test or a Chi-square test was used to compare frequencies. Independent sample t-test or Mann-Whitney U test was used to compare continuous variables according to the distribution of data. Logistic regression was used for multivariable analysis and corrected for the potential inter-relationships between the variables. Receiver operating characteristic (ROC) tables were used to find the most accurate cut-off values for all imaging variables to distinguish between the benign vs s-HCC groups. Variables were then transformed into binary measures based on these cut-off values. The final multivariable logistic model was developed using these categorical measures; the predictive value of this model was tested using the Area Under the Receiver operating characteristic (AU-ROC) curve for logistic models. *P-values <*0.05 were considered statistically noteworthy in all analyses.

## Results

**Figure 1:**
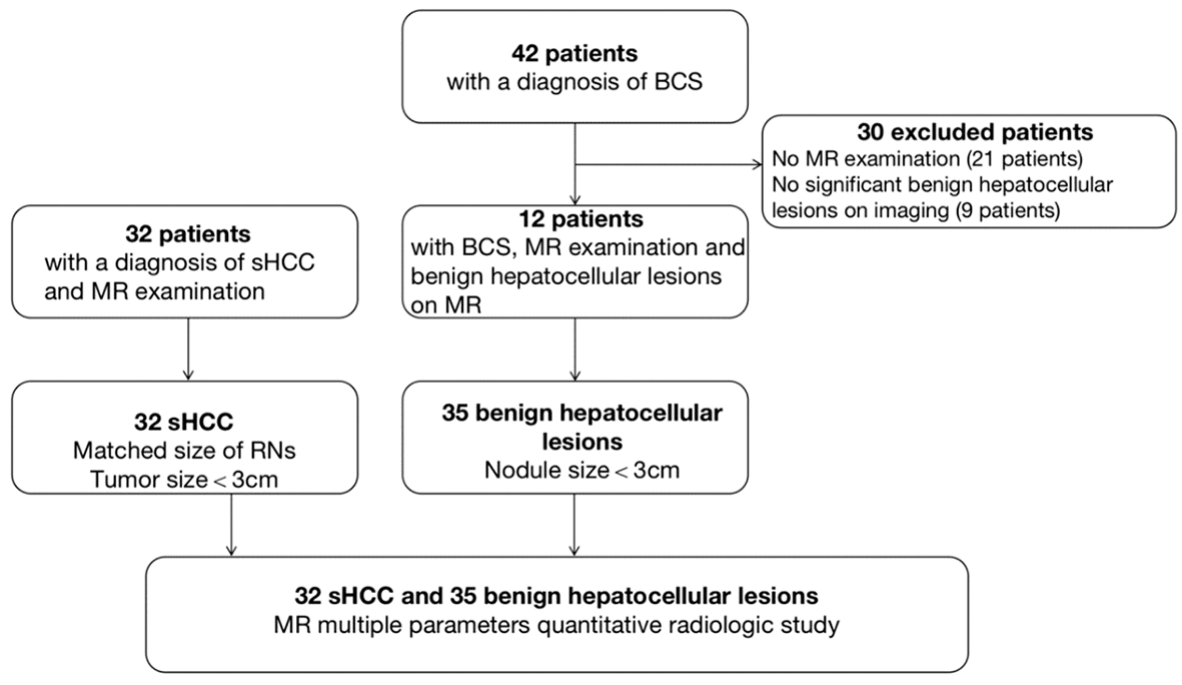
Flow chart of the study. BCS, Budd-Chiari syndrome; s-HCC, Small Hepatocellular Carcinoma.

A total of 35 benign hepatocellular lesions (n=12 patients) and 32 s-HCC lesions (n=32 patients) were considered for further analysis based on the inclusion criteria (**Figure 1**). In the benign cohort, the benign nature of 4 lesions was confirmed by histopathology (4/35), following indeterminate imaging characteristics; the other 31 lesions were identified as benign based on the stability of size and imaging features at least one year (median follow-up 60 months [12–168]) and AFP values less than 15 ng/ml. In the s-HCC cohort, the presence of disease was confirmed by histopathology in 14 lesions (14/32, 43.8%); the other 18 s-HCC lesions were confirmed based on the typical imaging features together with an elevated serum AFP level (**Figure 1**).

The demographic features of the patients are summarized in **Table 1**. There was no significant difference in the gender and age of the patients between the two groups. Serum AFP >15 ng/ml was observed in 19/32 (59%) patients with HCC versus 0/12 in the benign group (p <0.001). The median size of the lesions was similar between the groups (p=0.135), 1.76 cm in the benign group (IQR: 1.38-2.29 cm) and 1.95 cm in the s-HCC group (IQR: 1.75–2.36 cm). Washout was observed more frequently in s-HCC (22/32, 68.8%) than in benign liver lesions (n = 4/35, 11.4%, p<0.001); in contrast, a central scar was observed more frequently in benign lesions compared to HCC (70% vs 40%, p<0.001) (**Table 1**). We reported IQR, a measure of data spread, in our results since it offers robustness to outliers and helps characterize variability within the lesions, which can be essential for accurate diagnosis.

**Table 1:** Basic characteristics of benign hepatocellular lesions and s-HCC groups.

Characteristics	s-HCC (n= 32)	Benign lesions (n= 35)	*p-value*
**Age (year)**	67 ± 7.6	45 ± 15.1	<0.001
Sex			0.002
**Male**	27 (84%)	4 (33%)	
**Female**	5 (16%)	8 (67%)	
**AFP>15 ng/ml**	19/32 (59%)	0/12 (0%)	<0.001
**Venous washout**	22/32 (69%)	4/35 (11%)	<0.001
**Central Scar**	1/32 (3%)	14/35 (40%)	<0.001
**Lesion Size (cm)**	1.95 (1.75-2.36)	1.76 (1.38-2.29)	0.135

### Univariate analysis and cut-off values

**Figure 2:**
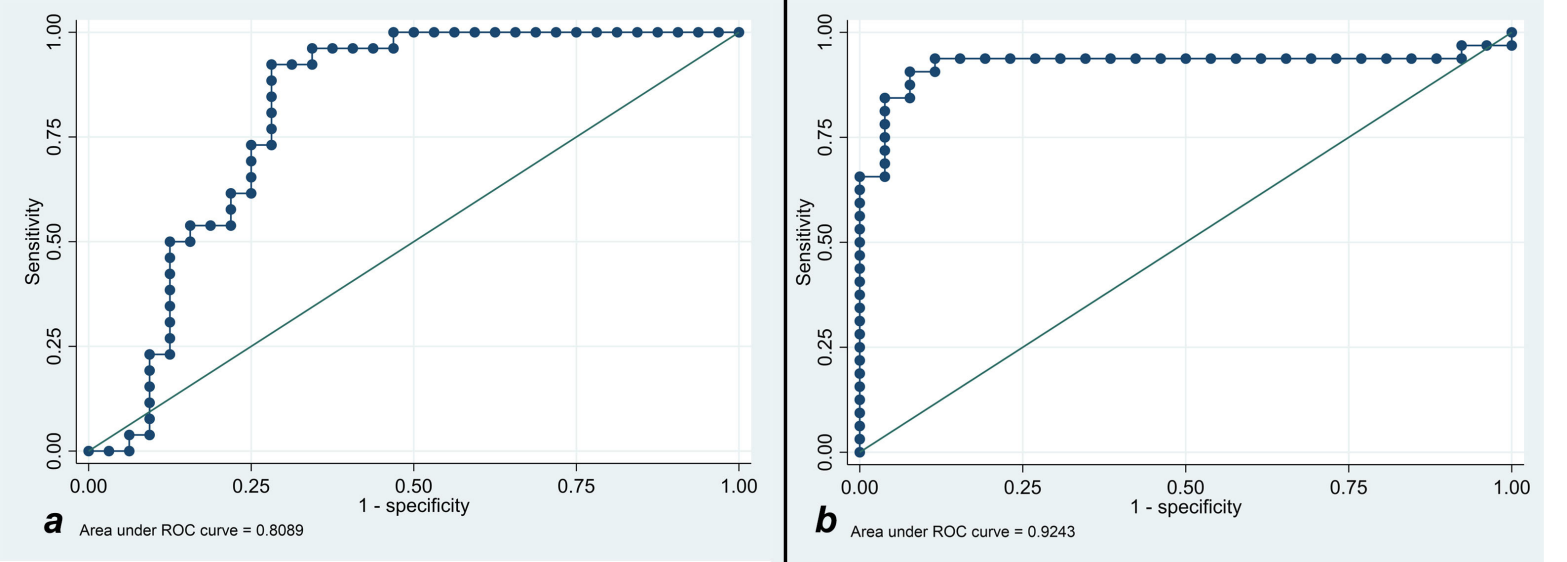
**(A)** Receiver operating characteristic curve analysis for showing T1 cut-off discriminatory to prognose the benignity of the lesion. **(B)** Receiver operating characteristic curve analysis for showing T2 cut-off discriminatory to prognose the malignancy of the lesion.

Lesion and liver signal intensity and ADC values, as well as lesion to liver percentages, are summarized in **Table 2**. There was no significant difference in T1SI, T2SI, and ADC values between s-HCC and benign lesions (p=0.069, p=0.359, p=0.236, respectively). However, after correcting the signal intensity based on the background liver signal (e.g. T1_lesion_/T1_liver_), a significant difference was observed between the lesion-to-liver ratios of T1 and T2 (T1-ratio and T2-ratio, respectively). S-HCC lesions demonstrated a lower T1-ratio versus benign lesions (medians 0.86 [IQR: 0.79-1.14] vs 1.25 [IQR: 1.14- 1.42]; *p*<0.001). A T1-ratio cut-off value of 1.05 yielded an 81.03% correct classification rate between the two groups, with a sensitivity of 92.3% and specificity of 71.9%. The area under the ROC curve for T1-ratio was 0.81, demonstrating a good discriminatory power (**Figure 2A**).

**Table 2:** Summary statistics for the parameters in the patients with benign hepatocellular lesions in Budd-Chiari syndrome and s-HCC.

	s-HCC (n=32)	Benign lesions (n=35)	*p-value*
**T1 _liver_ (ms)**	193.89 (109.26-703.01)	335.69 (276.87-361.61)	0.399
**T1 _lesion_ (ms)**	214.57 (116.26-556.14)	423.49 (370.69-525.79)	0.069
**T2 _liver_ (ms)**	80.73 (48.60-253.90)	226.87 (153.97-229.84)	0.006
**T2 _lesion_ (ms)**	132.98 (78.47-292.56)	197.04 (115.94-224.46)	0.359
**T1 _lesion_/T1 _liver_**	0.86 (0.79-1.14)	1.25 (1.14-1.42)	<0.001
**T2 _lesion_/T2 _liver_**	1.49 (1.29-2.01)	0.88 (0.73-0.96)	<0.001
**ADC _lesion_ (×10^-6^ mm^2^/s)**	1261.64 ± 383.15	1117.94 ± 384.50	0.236
**ADC _lesion_/ADC _liver_**	0.98 ± 0.17	1.08 ± 0.18	0.045
**(A _Enh_-A _liver_)/A _liver_**	0.29 (0.13-0.62)	0.29 (0.15-0.48)	0.836
**(V _Enh_-V _liver_)/V _liver_**	-0.06 (-0.15-0.17)	0.35 (0.06-0.67)	<0.001
**(D _Enh_-D _liver_)/D _liver_**	-0.06 (-0.17-0.09)	0.17 (0.05-0.26)	<0.001

An _Enh_, Arterial phase enhancement of lesion; A _liver_, Arterial phase enhancement of liver parenchyma; V _Enh_, Venous phase enhancement of lesion; V _liver_, Venous phase enhancement of liver parenchyma; D _Enh_, Delayed phase enhancement of lesion; D _liver_, Delayed phase enhancement of liver parenchyma.

T2-ratio was higher in s-HCC compared with benign lesions, (medians 1.49 [IQR: 1.29-2.01] vs 0.88 [IQR: 0.73-0.96]; p<0.001). A T2-ratio cut-off value of 1.07 had a 91.4% correct classification rate, with a sensitivity of 93.8% and specificity of 88.5%. The area under the ROC curve for T2-ratio was 0.92, with excellent discriminatory power between the two groups (**Figure 2B**).

The ADC-ratio (ADC_lesion_/ADC_liver_) was lower in s-HCC compared with the benign group (means 0.98 ± 0.17 vs 1.08 ± 0.18, *p*=0.045); however, the area under the ROC curve of 0.68 had poor discriminatory power and no discrete cut-off value was identified to have a reliable performance in differentiating between the two groups.

**Figure 3:**
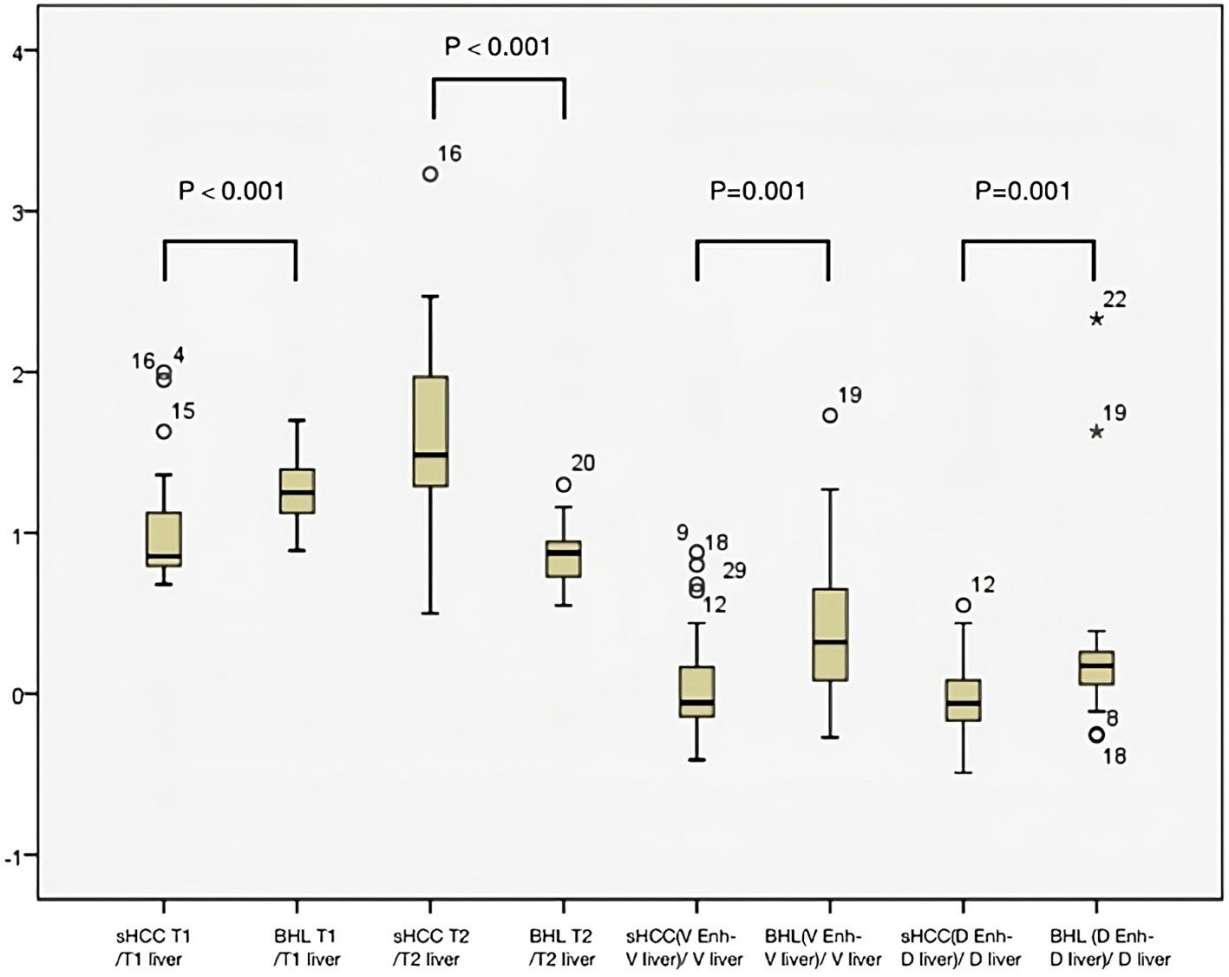
Box plot graph to measure the level of dispersion within each dataset. Mann-Whitney U test showed statistical significance between the benign hepatocellular lesions (BHL) group and s-HCC group for T1_lesion/_T1_liver_ and T2_lesion/_T2_liver_ (p<0.001 for both groups). The two groups differed statistically significantly from one another in the venous phase and the delayed phase of lesion enhancement relative to hepatic parenchyma (p <0.001 for both groups).

The arterial enhancement-ratio [(T1_lesion_- T1_liver_)/T1_liver_] in the s-HCC group was comparable with the benign lesion group (medians 0.29 [IQR: 0.13-0.62] vs 0.29 [IQR: 0.15-0.48]) (p = 0.836). The portal venous enhancement ratio was significantly lower in the s-HCC group versus the benign group (medians -0.06 [IQR: -0.15-0.17] vs 0.35 [IQR: 0.06- 0.67]) (p=0.001). Similarly, the s-HCC group demonstrated a lower delayed enhancement ratio versus the benign group (medians 0.06 [IQR -0.17-0.09] vs 0.17 [IQR: 0.05-0.26]) (p = 0.001) (**Figure 3**).

A cut-off value of 0.11 for the venous enhancement ratio showed a fair discriminatory power (AUC 0.73) with a sensitivity of 74.29% and specificity of 71.88%. Similarly, a cut-off value of 0.04 for the delayed enhancement ratio had fair discriminatory power (AUC 0.74, sensitivity 78.8%, specificity 71.9%).

All the imaging variables were transformed into binary measures based on the cut-off values. **Table 3** notes the bivariate correlations between these binary parameters and the diagnosis of s-HCC, in which T1-ratio, T2-ratio, portal venous, and delayed phase enhancement ratios demonstrated a significant correlation.

**Table 3:** Univariate correlations between the binary imaging parameters with benign hepatocellular lesions or s-HCC groups.

	n (%), total=67	s-HCC (n=32)	Benign hepatocellular lesion (n=35)	*p-value*
**T1-ratio > 1.05**	42 (63%)	9 (22%)	33 (78%)	<0.001
**T2-ratio > 1.07**	42 (62%)	30 (72%)	12 (28%)	<0.001
**PVP-Enh. ratio > 0.11**	31 (46%)	7 (23%)	24 (77%)	<0.001
**DP-Enh. ratio > 0.04**	36 (54%)	9 (25%)	27 (75%)	<0.001

s-Hcc, small Hepatocellular Carcinoma; PVP-Enh, Portal Venues Phase Enhancement; DP-Enhancement, Delayed Phase Enhancement.

### Multivariable analysis and final predictive model

**Figure 4:**
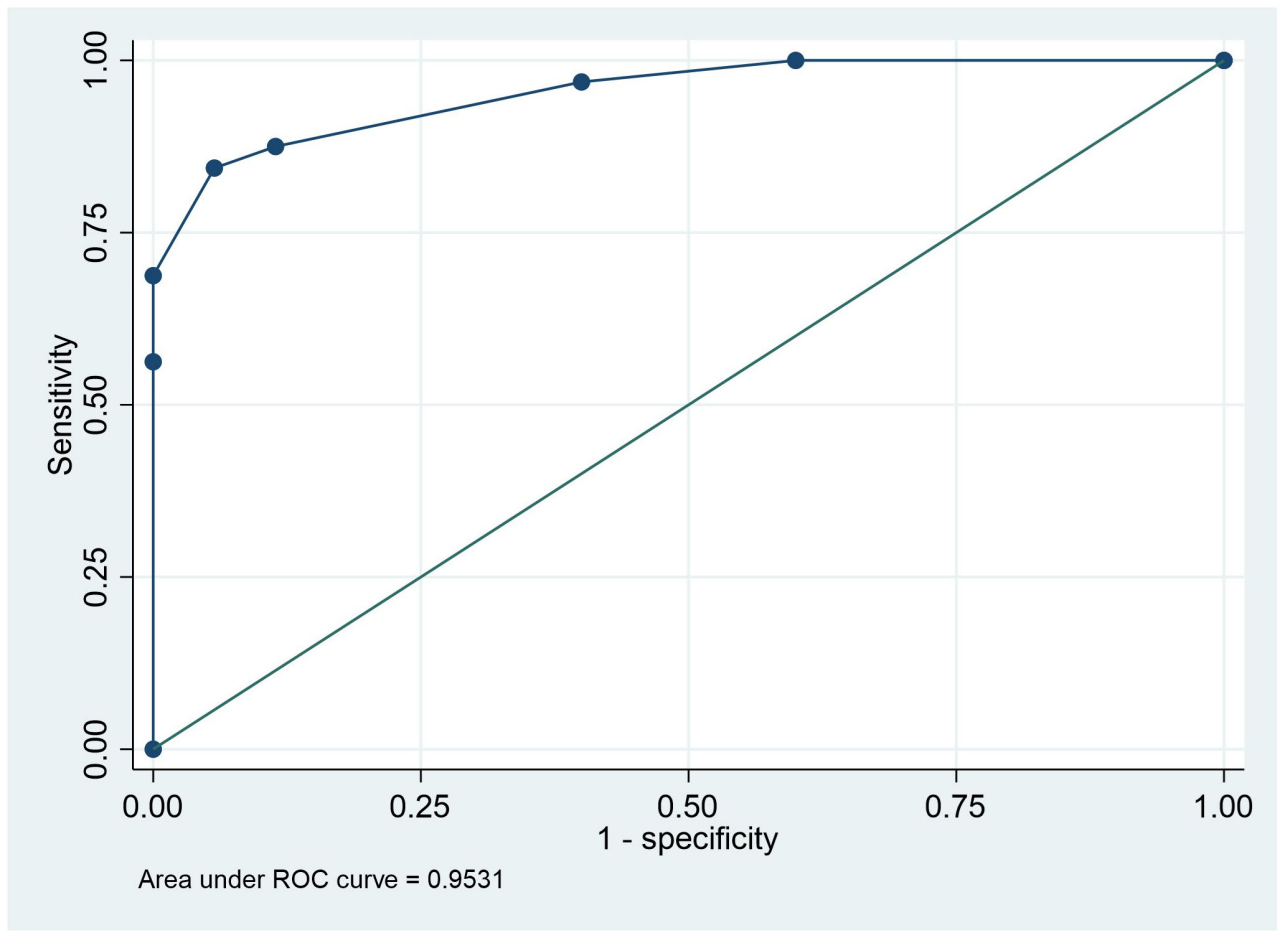
Receiver operating characteristic curve analysis indicating the power of multivariant logistic model analysis based on T1 ratio binary, T2 ratio binary, and PVP ratio binary to predict the lesion prognosis.

The initial multivariable model was developed using the quantitative variables with significant p-values in univariate analysis, considering the inter-relationships between the parameters. T1-ratio, T2-ratio, and portal venous enhancement ratio retained their predictive performance; hence, ADC-ratio lost its significant correlation and was eliminated from the final predictive model (**Table 4**). The delayed enhancement ratio was outperformed by the portal venous enhancement ratio. The ROC analysis for the logistic model demonstrated an excellent discriminatory power between the two groups, with the area under the curve of 0.94. Replacing the binary form of imaging variables into the final model, further improved the predictive performance of the model with area under the ROC-curve of 0.95, showing an excellent predictive power (**Figure 4**). Detailed odds ratios and p-values of the final predictive models are presented in **Table 4**.

**Table 4:** Multivariable logistic model for differentiating s-HCC from benign lesions.

	Odds ratio	p-value	95% confidence interval
**T1-ratio**	0.01	0.004	0.001-0.260
**T2-ratio**	564.84	0.003	8.460-37715.850
**PVP-Enh. ratio**	0.36	0.001	0.195-0.654
The area under ROC curve 0.9388			
**T1-ratio binary**	0.037	0.002	.0046888.2943348
**T2-ratio binary**	71.87	0.005	3.702021 1395.441
**PVP-Enh. ratio**	0.11	0.018	.0181999.6863284
The area under ROC curve 0.9531			

s-Hcc, small Hepatocellular Carcinoma; PVP-Enh, Portal Venues Phase Enhancement.

## Discussion

In this study, we identified specific MR imaging features to distinguish s-HCC from benign hepatocellular lesions in BCS patients. T1 and T2 of lesion/liver ratios were notably different between the two groups, with s-HCC having a lower T1 ratio and higher T2 ratio, compared with benign hepatocellular lesions. Following contrast administration, the venous enhancement ratio was lower for s-HCC than for benign hepatocellular lesions, and the difference was statistically significant. Multivariable analysis revealed that the model that included T1-ratio, T2-ratio, and portal venous enhancement ratio had a ROC of 0.94 to distinguish s-HCC from benign hepatocellular lesions. Using threshold values of 1.05, 1.07, and 0.11 for the T1 ratio, T2 ratio, and venous enhancement ratio respectively, for the multivariable analysis yielded a slight improvement in ROC of 0.95 to distinguish between the 2 groups.

**Figure 5:**
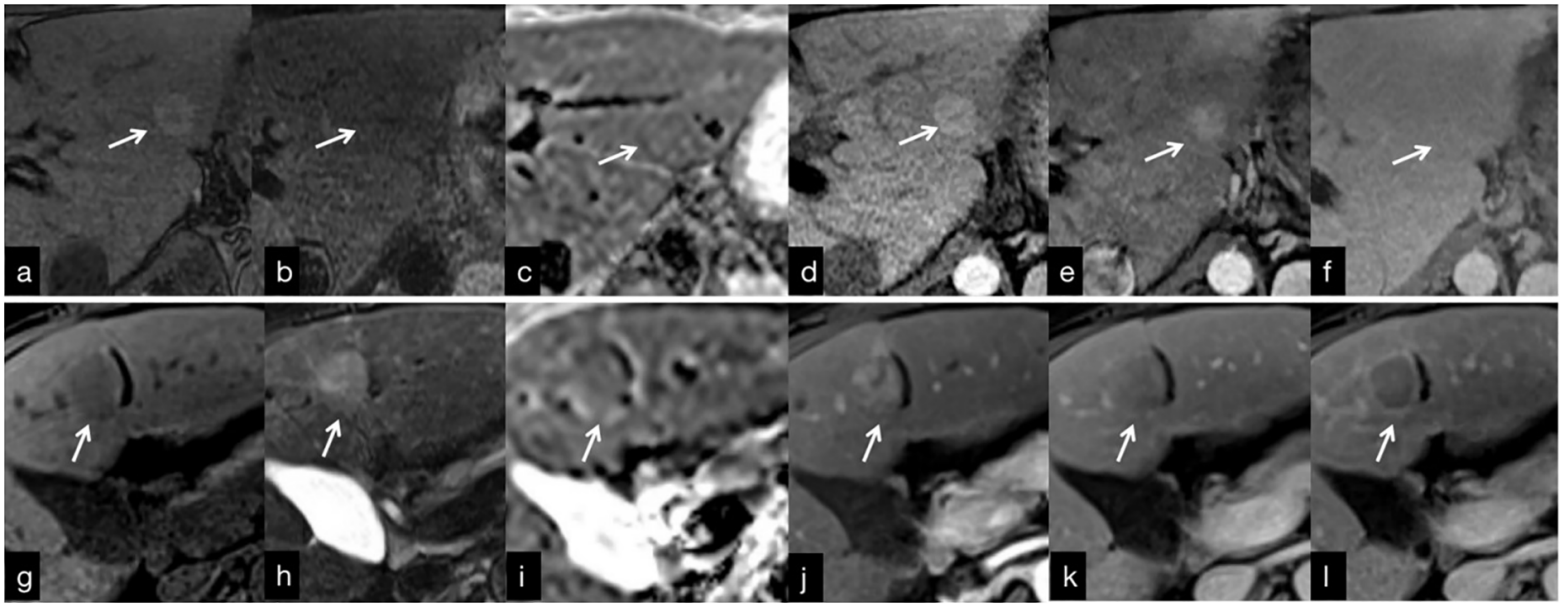
**(A–F)** MRI findings in a 30-year-old man with Budd-Chiari syndrome. **(A)** There was an RN in segment 2 (arrow) that is hyperintense on T1WI, with a hypointense central scar. **(B)** T2WI shows a homogeneous slightly hypointense lesion. **(C)** On the ADC map, the lesion is slightly hyperintense. **(D)** The arterial phase image shows homogeneous enhancement that persists in the venous **(E)** and delayed **(F)** phases. g-f: MRI findings in a 61-year-old woman with hepatocellular carcinoma. There is a s-HCC in segment 4 (arrow). **(G)** T1WI shows a hypointense lesion that is slightly hyperintense on T2WI **(H)**. **(I)** ADC map shows slightly hypointense lesion. The lesion is hypervascular in the arterial phase **(J)**, with washout in the venous **(K)** and delayed **(L)** phases.

**Figure 6:**
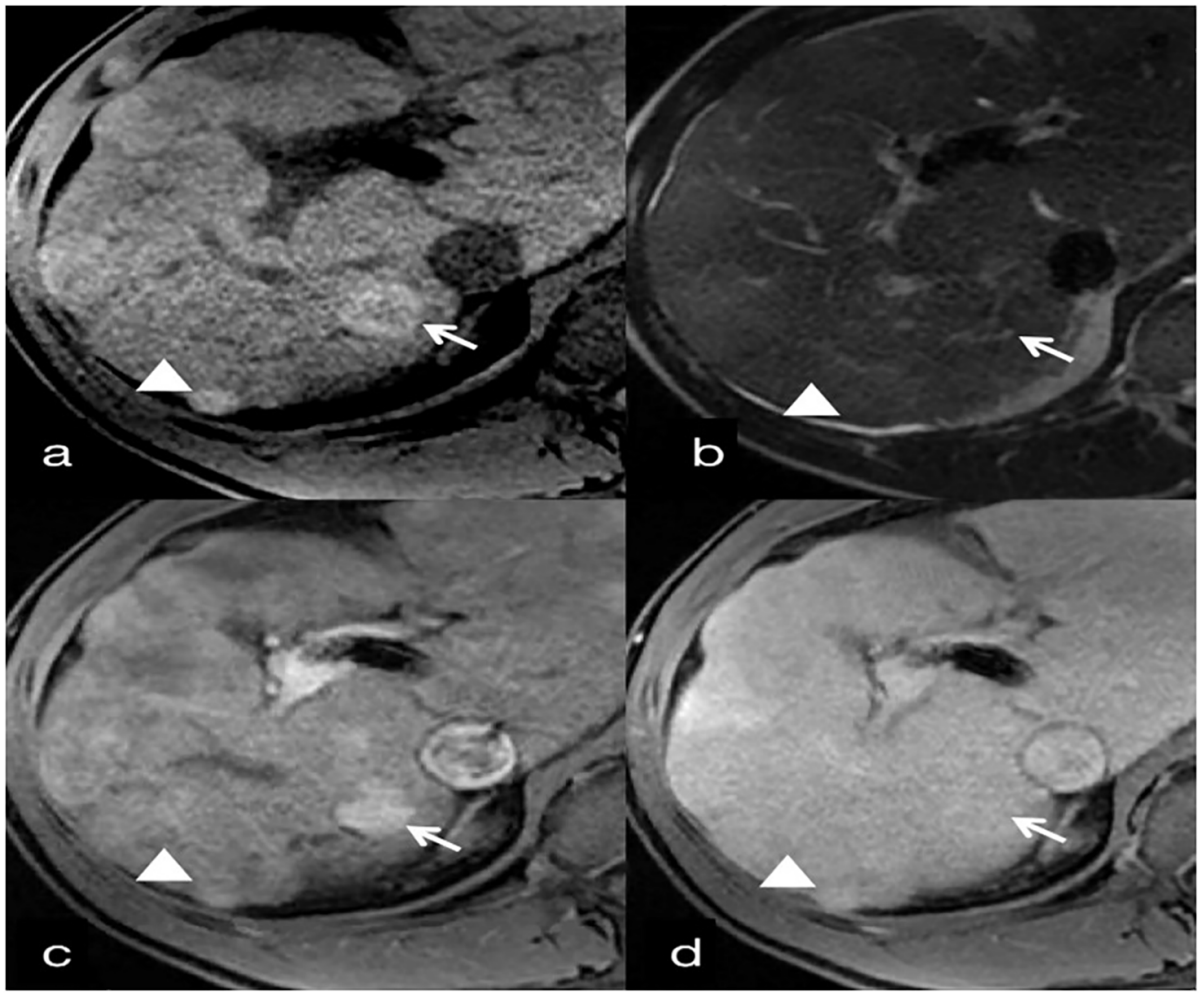
30-year-old man with benign hepatocellular lesions associated with Budd-Chiari syndrome. **(A)** T1-weighted image shows hyperintense benign hepatocellular lesions (arrow) with an internal hypointense scar. A second benign hepatocellular lesion (<2cm) under the liver capsule is homogeneously hyperintense (arrowhead). **(B)** T2-weighted image shows mildly hypointense benign hepatocellular lesions compared to surrounding liver parenchyma, with an internal small hyperintense scar (arrow). A second benign hepatocellular lesion (<2cm) is homogeneously mildly hypointense (arrowhead). **(C)** Contrast-enhanced T1-weighted image obtained 20 sec (arterial phase) after initiation of contrast agent administration shows homogeneous hyperenhancement of both lesions, which are slightly hyperintense in the venous phase **(D)**.

Budd-Chiari syndrome (BCS) is associated with a significant increase in the annual hospitalization rate in the United States, with myeloproliferative disorders and hypercoagulable states being the most prevalent risk factors. In BCS patients who develop hepatocellular carcinoma, hepatitis C infection has been identified as a potential predisposing factor (12). However, the process of cirrhosis related to chronic liver congestion is considered a prominent contributing factor (6, 13). Differentiating benign lesions from hepatocellular carcinoma is crucial in the management of BCS patients presenting with a lesion in the liver but continues to be challenging. On non-contrast MRI, most benign hepatocellular lesions in BCS patients appeared homogeneously or peripherally hyperintense relative to the background liver on T1-weighted imaging (T1WI), while s-HCC tended to be homogeneously hypointense (**Figures 5A, G**). The values are higher than the cutoff point of 1.042 and were suggestive of benign hepatocellular lesions based on our analysis. The exact reason for the hyperintensity of benign hepatocellular lesions on T1WI remains unclear, but it may be related to the lower signal intensity of the congested peri nodular area. Previous studies have noted that benign hepatocellular lesions in BCS closely resemble focal nodular hyperplasia (FNH) on gross and microscopic evaluation (14). Additionally, large lesions with a central scar have been found to share pathological similarities with FNH (15). In our study, A central scar was frequently seen in lesions larger than 2 cm in diameter on T1WI, while homogeneous hyperintensity was more commonly observed in benign hepatocellular lesions smaller than 2 cm (**Figure 6**). The presence of a central scar may serve as a distinctive finding in benign hepatocellular lesions larger than 2 cm in BCS patients (**Figure 6**), as suggested in previous research (6).

Most benign hepatocellular lesions in BCS patients appeared homogeneous or peripherally hypointense relative to the background liver on T2-weighted imaging (T2WI), whereas s-HCC exhibited homogeneous hyperintensity (**Figures 5H**). The majority of benign hepatocellular lesions detected on T2WI were relatively hypointense (85.7%, 30 of 35 lesions) (**Figures 5B, D**). It should be noted, however, that not all benign hepatocellular lesions in BCS demonstrated hypointensity on T2WI. Values higher than 1.071 were considered s-HCC based on our analysis with a sensitivity of 93.75% and a specificity of 88.46%. Small hepatocellular carcinomas arising in BCS patients may also exhibit hypointensity on T2WI when the surrounding liver parenchyma is severely congested. Therefore, relying solely on this result might not rule out the potential of cancer, necessitating a multiparametric approach.

In terms of diffusion-weighted imaging (DWI), benign hepatocellular lesions in BCS patients demonstrated higher ADC values relative to the background liver parenchyma compared with s-HCC (p=0.045). We considered lesions with values more than 1.038 as benign lesions with weak sub-optimal accuracy. However, the distinction between these lesions based on ADC values alone may be challenging (**Figures 5C, I**). Interestingly, combining T1 and T2 signal intensity with ADC values proved to be helpful in discriminating between benign hepatocellular lesions and s-HCC, offering a potential means for characterizing and monitoring focal lesions in BCS patients without the need to utilize contrast agents.

Contrast-enhanced MRI plays a crucial role in the evaluation of hepatocellular lesions. Among BCS patients, benign hepatocellular lesions typically exhibit hyperenhancement in the arterial phase and homogeneous hyperenhancement in the venous and delayed phases (**Figures 5D–F**). On the other hand, s-HCC frequently demonstrates heterogenous hyperenhancement in the arterial phase and hypoenhancement in the venous and delayed phases (**Figures 5J–L**). Therefore, distinguishing s-HCC from benign hepatocellular lesions based solely on arterial phase findings can be challenging, as both lesion types exhibit hypervascularity. However, the underlying mechanisms of arterial phase hyperintensity differ between benign hepatocellular lesions and s-HCC. While benign hepatocellular lesion hyperintensity on arterial phase T1-weighted imaging is not solely due to hypervascularity, previous studies have suggested the role of copper deposits or peri nodular parenchymal congestion in this phenomenon (12, 16). Conversely, s-HCC exhibits arterial phase hyperintensity solely due to hypervascularization. Our findings suggest that the presence of hypervascularity in the arterial phase may not be a reliable parameter for differential diagnosis (p=0.836). Of note, the current analysis did not show any specific cut-off for this phase of contrast enhancement. Furthermore, we observed that a significant difference between BCS patients with benign hepatocellular lesions and those with s-HCC occurred in the venous and delayed phases, where s-HCC exhibited contrast washout, indicative of malignant features (**Figures 5K, L**). While it is worth noting that a few benign hepatic lesions can also demonstrate washout, the frequency of washout was higher in s-HCC compared with benign lesions (68.8% vs. 11.4%, p <0.001) (3). Overall, contrast-enhanced MRI characteristics in the venous and delayed phases of s-HCC differ significantly from benign hepatocellular lesions in BCS patients. The data demonstrated that in the venous and delayed phase, values higher than 0.1144 and 0.0437, respectively, were more likely to be benign.

Our study had several limitations. Histopathological confirmation was not available for all patients with benign hepatocellular lesions. Performing histopathological examinations on all lesions was not clinically feasible, however. Rather, the stability in size and signal intensity of benign hepatocellular lesions during the one-year follow-up acted as some patients’ reference benchmark. In addition, the investigation was carried out at just one institution, resulting in a relatively small cohort of BCS patients. Nonetheless, inter-reader agreement for measuring the signal characteristics of hepatocellular lesions was excellent. However, it is important to note that in our study, one radiologist was responsible for placing the ROIs, which represents another limitation.

Future multicenter investigations will be required to confirm the precision and repeatability of these numerical measurements. Of note, no BCS patients in the current study developed s-HCC. Therefore, it remains unclear how the MRI imaging features would differ in s-HCC with and without BCS. Long-term imaging follow-up is warranted to evaluate the MRI imaging features of s-HCC in BCS patients.

In conclusion, we have identified specific MR imaging features that can help differentiate between benign hepatocellular lesions and s-HCC in patients with BCS.

## Data availability statement

The original contributions presented in the study are included in the article/supplementary material, further inquiries can be directed to the corresponding author.

## Ethics statement

The studies involving humans were approved by Johns Hopkins Hospital and University Medicine IRB. The studies were conducted in accordance with the local legislation and institutional requirements. Written informed consent for participation was not required from the participants or the participants’ legal guardians/next of kin in accordance with the national legislation and institutional requirements.

## Author contributions

GZ: Writing – review & editing, Formal analysis, Validation, Writing – original draft. HS: Writing – review & editing. HT: Writing – review & editing, Data curation, Methodology. QW: Writing – review & editing. MS: Writing – review & editing, Formal analysis. BH: Writing – review & editing. AB: Writing – review & editing. SA: Writing – review & editing. FV: Writing – review & editing. TP: Writing – review & editing. IK: Writing – review & editing, Conceptualization, Supervision.
